# The Role of Thromboelastography in Identifying Coagulopathy Among Geriatric Traumatic Brain Injury Patients

**DOI:** 10.7759/cureus.32818

**Published:** 2022-12-22

**Authors:** Alexander M Busko, Joshua J Solano, Lisa M Clayton, Patrick G Hughes, Richard J Paley, Richard D Shih, Scott M Alter

**Affiliations:** 1 Critical Care, University of Alabama at Birmingham, Birmingham, USA; 2 Department of Emergency Medicine, Florida Atlantic University Charles E. Schmidt College of Medicine, Boca Raton, USA; 3 Emergency Department, St Mary’s Medical Center, West Palm Beach, USA

**Keywords:** intracranial hemorrhage (ich), anti-coag, geriatric injuries, anticoagulation reversal, thromboelastography

## Abstract

Background

The geriatric population has the highest incidence of head injury, and those who are anticoagulated have an increased risk of traumatic intracranial hemorrhage (ICH). The availability of viscoelastic coagulation studies has coincided with the development of many anticoagulation reversal agents. In this study, our objective was to assess whether the thromboelastography (TEG) assay affected clinical decision-making regarding reversal agent administration among geriatric patients with ICH caused by blunt head trauma.

Methodology

We prospectively screened adults aged 65 and older with head trauma presenting to the emergency departments of two level-one trauma centers. International Classification of Diseases, Tenth Revision codes S00-09 were used to identify the diagnosis of head injury. Patients with CT head imaging positive for acute ICH were included. Each patient was assessed for home use of antiplatelet or anticoagulant medications, as well as in-hospital use of any reversal agents. Reversal agent administration and mortality were compared between patients who received TEG and those who did not.

Results

A total of 680 patients had acute ICH on head CT, and 324 (48%) patients received TEG. More patients screened with TEG were transfused platelets (30.2% vs. 10.7%, p < 0.001). This remained significant for patients taking anticoagulants, antiplatelets, or neither. There were no differences in the administration of other reversal agents (prothrombin complex concentrate or fresh frozen plasma) or mortality whether or not TEG was performed.

Conclusions

Patients who had TEG performed were more likely to receive platelet reversal agents, regardless of antiplatelet medication usage. Among elderly adults with ICH, TEG is a rapid screening test that may help identify patients with platelet function abnormalities requiring reversal.

## Introduction

The American population is aging rapidly. Over the next 40 years, the number of Americans older than 65 years is projected to nearly double. By 2060, the number of Americans living beyond 85 years will triple, and the United States is expected to add half a million centenarians [1]. Elderly adults have the highest incidence of traumatic brain injury (TBI), and this too is expected to grow along with the aging population, placing an enormous burden on the US healthcare system [2,3].

As life expectancy continues to rise, older adults are also increasingly likely to be prescribed antiplatelet and anticoagulant medications to manage or prevent thromboembolic disease. Although there is little data quantifying just how many Americans are prescribed these medications, it has been estimated that over eight million are on anticoagulation such as warfarin and direct-acting oral anticoagulants (DOAC) [4]. The number of Americans on single and dual antiplatelet therapy is likely several times that number.

Thromboelastography (TEG) is a viscoelastic coagulation assay that has become widely available in trauma centers over the last decade. Traditional coagulation studies (prothrombin time (PT)/international normalized ratio (INR) and activated partial thromboplastin time (aPTT)) measure clotting factor activity and only describe the time to thrombus initiation. TEG provides quantitative measurements of thrombus initiation, propagation, strength, and lysis. Abnormal values can be used to guide the transfusion of agents such as fresh frozen plasma (FFP), platelets, and cryoprecipitate to correct specific coagulopathies [5].

We hypothesized that TEG would influence clinical decision-making surrounding the use of reversal agents among a geriatric cohort of TBI patients with a high rate of anticoagulant and antiplatelet use.

## Materials and methods

Study design and setting

This was a prospective observational study conducted at two level-one trauma centers in South Florida, a geographic area with one of the largest geriatric populations in the United States [6]. This study follows the Strengthening the Reporting of Observational Studies in Epidemiology (STROBE) reporting guidelines. Institutional review board approval was obtained at the study hospitals and their affiliated university. All treatment decisions were at the sole discretion of the attending trauma surgeon without a specific protocol for care.

Selection of participants

All patients 65 years or older with blunt head injuries presenting to the emergency department between August 2019 and August 2020 were screened. International Classification of Diseases, Tenth Revision codes beginning with S00-09 were used to identify the diagnosis of TBI. Patients were included if they had CT head imaging positive for acute intracranial hemorrhage (ICH) and a TEG performed.

Measurements

Trained research assistants collected the following data from the electronic medical record: use of antithrombotic medications and which one(s), the performance of TEG, CT head results, use of reversal agents and which one(s), hospital disposition (discharge/hospice/expired), and hospital length of stay. Head CT results were reviewed by the physician investigators to determine whether the CT finding indicated an acute ICH or not. As all data were collected from the electronic medical record during the patients’ initial hospitalization, there were no missing data and no loss to follow-up. The state death registry was queried to obtain 90-day mortality. Our primary outcome was the use of any reversal agent, with a secondary outcome of mortality.

Analysis

Reversal agent administration, in-hospital mortality, discharge to hospice, and 90-day mortality were compared using chi-square analysis between patients who received TEG assays and those who did not. The t-test was used for the comparison of hospital length of stay. Analyses were performed using SPSS version 27.0 (IBM SPSS Statistics for Windows, Armonk, NY, USA).

## Results

Prospective data were collected for 5,776 consecutive adult patients over 65 years of age who underwent evaluation for a blunt head injury. Only 10% (n = 549) were evaluated with TEG. A total of 680 patients with acute ICH were identified on their initial head CT, whose average age was 81.5 years (standard deviation (SD) = 8.5), and 47% were female. Of the patients with acute ICH, 324 (48%) underwent TEG evaluation, whose average age was 81.9 years (SD = 8.1), and 41% were female.

TEG was more likely to be performed on patients taking antiplatelet medication (60.1% vs. 36.1%, p < 0.001, odds ratio (OR) = 2.664, 95% confidence interval (CI) = 1.954 to 3.633), but not more likely to be performed on patients taking anticoagulant medication (47.7% vs. 47.6%, p = 0.985, OR = 1.003, 95% CI = 0.700 to 1.439). A total of 206 patients met the primary outcome of receiving any reversal agent. Patients who received a TEG were more likely to be administered a reversal agent than those who did not (39.5% vs. 21.9%, p < 0.001, OR = 2.328, 95% CI = 1.664 to 3.256).

Platelet transfusion was the most common reversal agent administered, with 136 (20.0%) patients receiving this therapy. Prothrombin complex concentrate (PCC) was given to 80 (11.8%) patients, and vitamin K was given to 34 (5.0%) patients. FFP, desmopressin (DDAVP), cryoprecipitate, and idarucizumab each were given to less than 2% of patients. Table 1 includes an example of a 94-year-old female patient on both aspirin and clopidogrel who received two units of platelets for an acute subdural hematoma.

**Table 1:** Thromboelastography values for a patient receiving platelets for an acute traumatic subdural hematoma. TEG = thromboelastography; LY30 = clot lysis at 30 minutes after maximum clot strength; ADP = adenosine diphosphate; AA = arachidonic acid; MA = maximal amplitude

TEG value	Patient’s value	Reference range
React time TEG	3.7 (L)	5.0–10.0
K speed TEG	0.8 (L)	1.0–3.0
Angle TEG	78.9 (H)	53.0–72.0
Maximal amplitude TEG	75.4 (H)	50.0–70.0
LY30	0.2	0.0–7.5
% inhibition ADP	28.1	No range given in the system
% inhibition AA	100.0	No range given in the system
MA (activated)	15.7	No range given in the system
MA (ADP)	58.6	No range given in the system
MA (AA)	14.9	No range given in the system

Among patients with acute ICH, those screened with TEG were more likely to receive a platelet transfusion (30.2% vs. 10.7%, p < 0.001). This association remained significant for patients taking antiplatelets, anticoagulants, or neither. Those screened with TEG were also more likely to be treated with DDAVP (1.2% vs. 0%, p = 0.035). There was no significant difference in the administration of FFP (1.5% vs. 1.7%, p = 0.883), PCC (12.0% vs. 11.5%, p = 0.833), vitamin K (4.9% vs. 5.1%, p = 0.944) or any of the other reversal agents whether or not patients were on anticoagulants and/or antiplatelets (Tables 2-4).

**Table 2:** Reversal agent administration by TEG performance. TEG = thromboelastography; OR = odds ratio; CI = confidence interval; DDAVP = desmopressin; FFP = fresh frozen plasma; PCC = prothrombin complex concentrate

	TEG (n = 324)	No TEG (n = 356)	OR (95% CI)	P-value
Platelets	98 (30.2%)	38 (10.7%)	3.63 (2.41-5.48)	<0.001
DDAVP	4 (1.2%)	0 (0%)	-	0.035
FFP	5 (1.5%)	6 (1.7%)	0.91 (0.28-3.03)	0.883
PCC	39 (12.0%)	41 (11.5%)	1.05 (0.66-1.68)	0.833
Vitamin K	16 (4.9%)	18 (5.1%)	0.98 (0.49-1.95)	0.944

**Table 3:** Reversal agent administration by TEG performance for patients on antiplatelets. TEG = thromboelastography; OR = odds ratio; CI = confidence interval; DDAVP = desmopressin; FFP = fresh frozen plasma; PCC = prothrombin complex concentrate

	TEG (n = 197)	No TEG (n = 131)	OR (95% CI)	P-value
Platelets	92 (46.7%)	31 (23.7%)	2.83 (1.73-4.62)	<0.001
DDAVP	3 (1.5%)	0 (0%)	-	0.156
FFP	2 (1.0%)	2 (1.5%)	0.66 (0.09-4.76)	0.679
PCC	20 (10.2%)	15 (11.5%)	0.87 (0.43-1.78)	0.709
Vitamin K	5 (2.5%)	4 (3.1%)	0.83 (0.22-3.14)	0.780

**Table 4:** Reversal agent administration by TEG performance for patients on anticoagulants. TEG = thromboelastography; OR = odds ratio; CI = confidence interval; DDAVP = desmopressin; FFP = fresh frozen plasma; PCC = prothrombin complex concentrate

	TEG (n = 73)	No TEG (n = 80)	OR (95% CI)	P-value
Platelets	19 (26.0%)	6 (7.5%)	4.34 (1.62-11.59)	0.002
DDAVP	1 (1.4%)	0 (0%)	-	0.294
FFP	4 (5.5%)	3 (3.8%)	1.49 (0.32-6.88)	0.609
PCC	39 (53.4%)	41 (51.2%)	1.09 (0.58-2.06)	0.788
Vitamin K	16 (21.9%)	18 (22.5%)	0.97 (0.45-2.08)	0.944

There was no difference in hospital mortality (2.8% vs. 2.8%, p = 0.980), discharge to hospice (14.5% vs. 9.8%, p = 0.062), or 90-day mortality (25.9% vs 23.3%, p = 0.429) among patients screened with TEG and those who were not (Table 5). Length of hospital stay was longer for patients who had a TEG performed versus those who did not (5.6 (SD = 5.8) vs 4.6 days (SD = 6.7), p = 0.035).

**Table 5:** Patient outcomes by TEG performance. TEG = thromboelastography; OR = odds ratio; CI = confidence interval

	TEG (n = 324)	No TEG (n = 356)	OR (95% CI)	P-value
In-hospital mortality	9 (2.8%)	10 (2.8%)	0.99 (0.40-2.46)	0.980
Discharge to hospice	47 (14.5%)	35 (9.8%)	1.56 (0.98-2.48)	0.062
90-day mortality	84 (25.9%)	83 (23.3%)	1.15 (0.81-1.63)	0.429

## Discussion

In this prospective observational study, geriatric patients with traumatic ICH were more likely to receive desmopressin and three times more likely to receive platelet transfusion if they had TEG performed as part of their workup regardless of any pre-injury antiplatelet or anticoagulant use. These findings confirm our hypothesis regarding reversal agents affecting platelets but did not support our hypothesis regarding reversal agents affecting coagulation. These results suggest that the use of TEG can influence clinical decision-making among geriatric patients with TBI. However, we did not detect a significant difference in any patient-oriented outcomes. These findings are likely significant for the platelet reversal agents but not the coagulation reversal agents because of the consensus surrounding the reversal of warfarin and DOACs in the event of life-threatening bleeding. Given the lack of evidence when it comes to antiplatelet reversal, it is likely that many physicians have come to rely on TEG to inform their decision of whether to reverse these medications because there is no other alternative strategy.

The use of viscoelastic coagulation assays such as TEG and rotational thromboelastogram has increased substantially over the last decade, and studies of their utility in acutely bleeding patients have been largely positive. One study reported a mortality benefit in penetrating trauma patients undergoing TEG-guided massive transfusion [7]. A 2016 randomized trial found that TEG-guided resuscitation was associated with decreased mortality when compared with resuscitation guided by conventional coagulation tests [8].

Numerous studies have reported that TEG decreases blood product use in acutely bleeding patients: cardiac surgical patients, patients with cirrhosis and coagulopathy undergoing invasive procedures, and TBI patients on DOACs [9-11]. The most recent guidelines published by the Eastern Association for the Surgery of Trauma found a similar trend among acutely bleeding trauma, surgical, and critically ill patients and recommended TEG be used routinely in those populations [12].

There is broad consensus across society guidelines recommending routine warfarin reversal with PCC in the event of major life-threatening bleeding [13-15]. For life-threatening bleeding in patients on DOACs, they recommend idarucizumab be administered to reverse dabigatran and andexxanet alfa for apixaban or rivaroxaban. In the event andexxanet alfa is unavailable, they generally recommend four-factor PCC as a second-line agent.

There are no evidence-based recommendations to guide antiplatelet reversal. The PATCH trial was a randomized controlled trial in patients on antiplatelet therapy who developed spontaneous ICH. In this study, patients treated with platelet transfusion had worse functional outcomes and increased in-hospital morbidity and mortality [16]. Other studies have reported mixed results on the effect of platelet transfusion on mortality but may have an impact on secondary outcomes [17,18]. Another study with limited data on 12 patients reported platelet mapping to be ineffective, but their conclusions are limited [19]. Unfortunately, there are no randomized controlled trials evaluating antiplatelet reversal strategies among TBI patients. Our study did not detect a significant difference in outcomes between patients treated with platelet transfusion and desmopressin and those who were not.

This study has several limitations that bear mention. First, there is the issue of spectrum bias. Our study was conducted at two level-one trauma centers. It is possible these high acuity centers produced a study population with more severe injuries than might have been seen at surrounding hospitals without level-one trauma designation. Furthermore, our study was conducted in a county with a large geriatric population. Such a disproportionate number of geriatric patients, many of whom are on anticoagulation or antiplatelets, could limit the external validity of our results. Second, there was no study protocol to determine when TEG would be performed or reversal agents administered. This was left up to the clinician’s judgment, and only 48% of patients with CT-confirmed ICH received a TEG. While it is standard practice at our institution to administer reversal agents to patients with life-threatening bleeding due to warfarin or DOACs, the same cannot be said of platelet reversal agents. All of these underscore the need for randomized trials addressing this question.

## Conclusions

In this prospective study, geriatric patients with traumatic ICH who had a TEG performed were more likely to receive platelet reversal agents, regardless of whether or not they are taking antiplatelet medications. This study shows an increased role for TEG in a population with anticoagulant and antiplatelet medication.

Among high-risk geriatric adults with traumatic ICH, TEG is a rapid screening test that may help identify patients with platelet function abnormalities requiring reversal. The effects of the TEG may contribute to changes in clinical management in this population. Given how prevalent antiplatelet therapy has become, randomized controlled trials are urgently needed to inform our treatment strategies.
